# Efficacy of a sensory deterrent and pipe modifications in decreasing entrainment of juvenile green sturgeon (*Acipenser medirostris*) at unscreened water diversions

**DOI:** 10.1093/conphys/cou056

**Published:** 2014-12-05

**Authors:** Jamilynn B. Poletto, Dennis E. Cocherell, Timothy D. Mussen, Ali Ercan, Hossein Bandeh, M. Levent Kavvas, Joseph J. Cech, Nann A. Fangue

**Affiliations:** 1 Department of Wildlife, Fish, and Conservation Biology, University of California, Davis, One Shields Avenue, Davis, CA 95616, USA; 2 Department of Civil and Environmental Engineering, University of California, Davis, One Shields Avenue, Davis, CA 95616, USA

**Keywords:** Conservation, entrainment, fish, green sturgeon, swimming performance, water diversion

## Abstract

Water projects designed to extract fresh water for local urban, industrial and agricultural use throughout rivers and estuaries worldwide have contributed to the fragmentation and degradation of suitable habitat for native fishes. The number of water diversions located throughout the Sacramento–San Joaquin watershed in California's Central Valley exceeds 3300, and the majority of these are unscreened. Many anadromous fish species are susceptible to entrainment into these diversions, potentially impacting population numbers. In the laboratory, juvenile green sturgeon (*Acipenser medirostris*) have been shown to have high entrainment rates into unscreened diversions compared with those of other native California fish species, which may act as a significant source of mortality for this already-threatened species. Therefore, we tested the efficacy of a sensory deterrent (strobe light) and two structural pipe modifications (terminal pipe plate and upturned pipe configuration) in decreasing the entrainment of juvenile green sturgeon (mean mass ± SEM = 162.9 ± 4.0 g; mean fork length = 39.4 ± 0.3 cm) in a large (>500 kl) outdoor flume fitted with a water-diversion pipe 0.46 m in diameter. While the presence of the strobe light did not affect fish entrainment rates, the terminal pipe plate and upturned pipe modifications significantly decreased the proportion of fish entrained out of the total number tested relative to control conditions (0.13 ± 0.02 and 0.03 ± 0.02 vs. 0.44 ± 0.04, respectively). These data suggest that sensory deterrents using visual stimuli are not an effective means to reduce diversion pipe interactions for green sturgeon, but that structural alterations to diversions can successfully reduce entrainment for this species. Our results are informative for the development of effective management strategies to mitigate the impacts of water diversions on sturgeon populations and suggest that effective restoration strategies that balance agricultural needs with conservation programmes are possible.

## Abstract (precis)

Water diversions pose a risk to passing fishes. We evaluated the effectiveness of several methods for preventing green sturgeon from being pulled into a simulated water diversion. We made recommendations for future management practices from the data that include considerations of fish sensory ecology as well as feasibility of implementation.

## Introduction

Throughout the world, increased water diversion by water projects (i.e. water pumping facilities, agricultural diversions, hydroelectric dams) from rivers and estuaries for human use is contributing to the loss of spawning habitat for native fishes (Sheer and Steel, 2006), heightened impedance to fish migration and movement (Larinier, 2001; Morita and Yamamoto, 2002; Dugan *et al.*, 2010) and the degradation of juvenile and adult habitat (Pelicice and Agostinho, 2008). Water diversion structures and water pumping activities can both directly influence the population numbers of fishes by causing mortality of adults or juveniles (Kimmerer, 2008; Baumgartner *et al.*, 2009), thereby affecting recruitment of a given spawning year class (Kimmerer, 2008; Grimaldo *et al.*, 2009), or indirectly by influencing local hydraulic and environmental conditions (Bunn and Arthington, 2002). For example, entrainment of fishes occurs when passing fish are drawn into the water diversion instead of remaining within the main water channel. Mortality occurs either due to the pumps and machinery or when fish are stranded in irrigation channels (Baumgartner *et al.*, 2009). In the Sacramento–San Joaquin watershed in central California, entrainment into water diversions has been linked to the declines of several native fish species, including species of particular conservation and management concern, such as delta smelt (*Hypomesus transpacificus*; Bennett, 2005) and green sturgeon (*Acipenser medirostris*; Mussen *et al.*, 2014a).

The effects of anthropogenic devices, such as water diversions, can pose greater risks to some native fishes than others, especially if species are already in decline or living in highly altered environments (Moyle, 2002). The green sturgeon, for example, is an anadromous fish species native to the Pacific coast of North America that is protected under the United States Endangered Species Act (ESA) of 2006. Green sturgeon have two distinct population segments (Israel *et al.*, 2004). The northern distinct population segment spawns primarily in the Rogue and Klamath rivers in Oregon, while the only known spawning locations of the southern distinct population segment are in the Central Valley of California. The southern distinct population segment is listed as ‘Threatened’ under the ESA. Due to their anadromous life history, juvenile green sturgeon migrate long distances from the upper reaches of the watershed to more estuarine waters within their first year of life (Beamesderfer *et al.*, 2006; Allen *et al.*, 2009), passing numerous water diversions in the outmigration process.

Sturgeon may be more susceptible to entrainment into water diversions, in part due to their reduced swimming capabilities compared with other fishes (Peake *et al.*, 1997). Green sturgeon, in particular, have a reduced critical swimming velocity when compared with salmonids (Peake *et al*., 1997), as well as with other species of sturgeon (Deslauriers and Kieffer, 2011), underscoring this deficiency. However, burst swimming capabilities are perhaps a more accurate measure of the ability of sturgeon to overcome the intake velocities at water diversions. Burst swimming capabilities have never been assessed in green sturgeon, but Peake *et al*. (1997), using swimming endurance data, inferred that lake sturgeon (*Acipenser fulvescens*) had a reduced burst swimming performance compared with salmonids. Likewise, juvenile shortnose (*Acipenser brevirostrum*) and Atlantic sturgeon (*Acipenser oxyrinchus*) showed a limited capacity to recover physiologically after exhaustive exercise compared with other teleosts, suggesting reduced burst swimming capabilities (Kieffer *et al*., 2001). Furthermore, juvenile green sturgeon begin to show an ontogenetic reduction in swimming performance abilities (a decrease in absolute Ucrit [maximum sustained swimming velocity]) around the size and age they begin their outmigration from freshwater rivers (∼25 cm total length), probably due to the energetic costs associated with physiological preparations for entry into saltwater (Allen *et al.*, 2006). Previous laboratory studies examining the risk of entrainment into open water diversion pipes suggest that juvenile green sturgeon are much more susceptible to entrainment than are Chinook salmon (*Oncorhynchus tshawytscha*; Mussen *et al.*, 2013, 2014a, b) and that water diversions pose a significant mortality risk for juvenile green sturgeon.

Direct mortality caused by entrainment into water diversions can be mitigated by fish protection devices (e.g. fish deterrents), which are used to prevent fish interactions with water-diversion structures. Many water projects are outfitted with guidance devices, such as louvre systems (vertically slatted metal grates), or physical barriers, such as fish-exclusion screens (Taft, 2000), that reduce fish entrainment (Gale *et al.*, 2008; Simpson and Ostrand, 2012; Boys *et al.*, 2013). While effective for some species, repeated contact with fish-exclusion screens can result in a heightened stress response (Young *et al.*, 2010), injury (Swanson *et al.*, 2004, 2005) or even subsequent mortality (Swanson *et al.*, 2005), particularly if fish become impinged on screen faces. Fish behaviour near exclusion screens is highly variable, and even closely related species can exhibit differential contact and impingement rates on screens (Poletto *et al.*, 2014). Furthermore, screen construction and installation can be very expensive, ranging from thousands to millions of dollars depending on the size of the diversion; these costs are frequently paid for by water diverters or state and federal agencies with cost-share programmes (McMichael *et al.*, 2004; Moyle and Israel, 2005). Maintenance of screens, particularly due to fouling and build-up of debris on screen faces (USBR, 2006), can also require thousands of dollars in annual effort (McMichael *et al.*, 2004). Indeed, of the more than 3300 water diversions that are located within the Sacramento–San Joaquin watershed, roughly 98% of them remain unscreened (Herren and Kawasaki, 2001), posing a significant threat to passing fish species.

An alternative to the installation of fish screens to reduce fish entrainment is the use of behavioural barriers, such as sensory deterrents (Taft, 2000). Sensory deterrents exploit the sensory systems of fishes to create stimuli that repel or prevent fish movement into a specific area (Noatch and Suski, 2012), and include the use of strobe lights, bubble curtains, sound generators, electric or magnetic fields, chemical cues or some combination of these (reviewed by USBR, 2006; Noatch and Suski, 2012). While certain applications have been successful at eliciting avoidance responses from some species of fish both in the field (Maes *et al.*, 2004; Hamel *et al.*, 2008) and in the laboratory (Kates *et al.*, 2012), others have proved unsuccessful (Johnson *et al.*, 2005; Poletto *et al.*, 2014). For example, previous laboratory studies on juvenile green and white sturgeon (*Acipenser transmontanus*) behaviour near fish screens in the laboratory suggest that strobe lights and mechanical vibrations are not effective at deterring interactions with diversion structures (Poletto *et al.*, 2014). However, strobe lights have not been tested for green sturgeon in a large-scale river simulation or in the field and should not be determined ineffective until more rigorous testing is completed. An alternative option is the use of structural modifications to the water-diversion pipes that result in alterations in water velocities and flow fields surrounding the intakes. Using laboratory investigations of entrainment into unscreened water diversions, we have previously shown that juvenile green sturgeon display a limited capacity for escaping entrainment flows (Mussen *et al.*, 2014a). Alterations to the intake velocities surrounding entrainment pipes, without changing the overall volume of water that is diverted, have potential to reduce green sturgeon entrainment. Coupled with a reduced intake velocity, physical modifications that alter flow cues may reduce the risk of entrainment of passing fishes.

The entrainment risk for juvenile green sturgeon posed by water diversions has the potential to exacerbate population declines and subvert conservation efforts for this species. Therefore, we investigated the efficacy of a commonly used sensory deterrent and two types of structural modifications designed to decrease the entrainment risk of juvenile green sturgeon. We used a large water flume outfitted with an ‘over-the-levee’ style water-diversion pipe to simulate conditions in the Sacramento River. The proportion of fish entrained through the pipe, entrainment risk, number of pipe passes and entrainment distances of fish were quantified in the presence of a strobe light deterrent, a terminal pipe-plate modification (TPP) and an upturned pipe modification (UTP) and compared with those of the unmodified pipe (control). We predicted that the strobe light sensory deterrent would not alter entrainment of green sturgeon, but that the two structural modifications (TPP and UTP) would significantly decrease the number of fish entrained and the entrainment risk compared with fish in the control conditions.

## Materials and methods

### Fish

Green sturgeon (F2, northern distinct population segment) were spawned from University of California Davis broodstock in April 2011 using previously established tank-spawning methodologies (Van Eenennaam *et al.,* 2001, 2012) and reared at the University of California Davis Center for Aquatic Biology and Aquaculture (CABA) at 18.5 ± 0.5°C in 815 litre round fibreglass tanks with continuous flows of aerated (dissolved oxygen 7.5 ± 1.0 mg O_2_ l^−1^), non-chlorinated fresh water from a dedicated well. Fish were fed continually to satiation with semi-moist commercial salmonid diet (Rangen, Inc., Buhl, ID, USA) and eventually weaned onto a dry pelleted diet (SilverCup™) at ∼60 days post-hatch. All handling, care and experimental procedures used were reviewed and approved by the University of California Davis Institutional Animal Care and Use Committee (IACUC #15836).

### Flume

**Figure 1:**
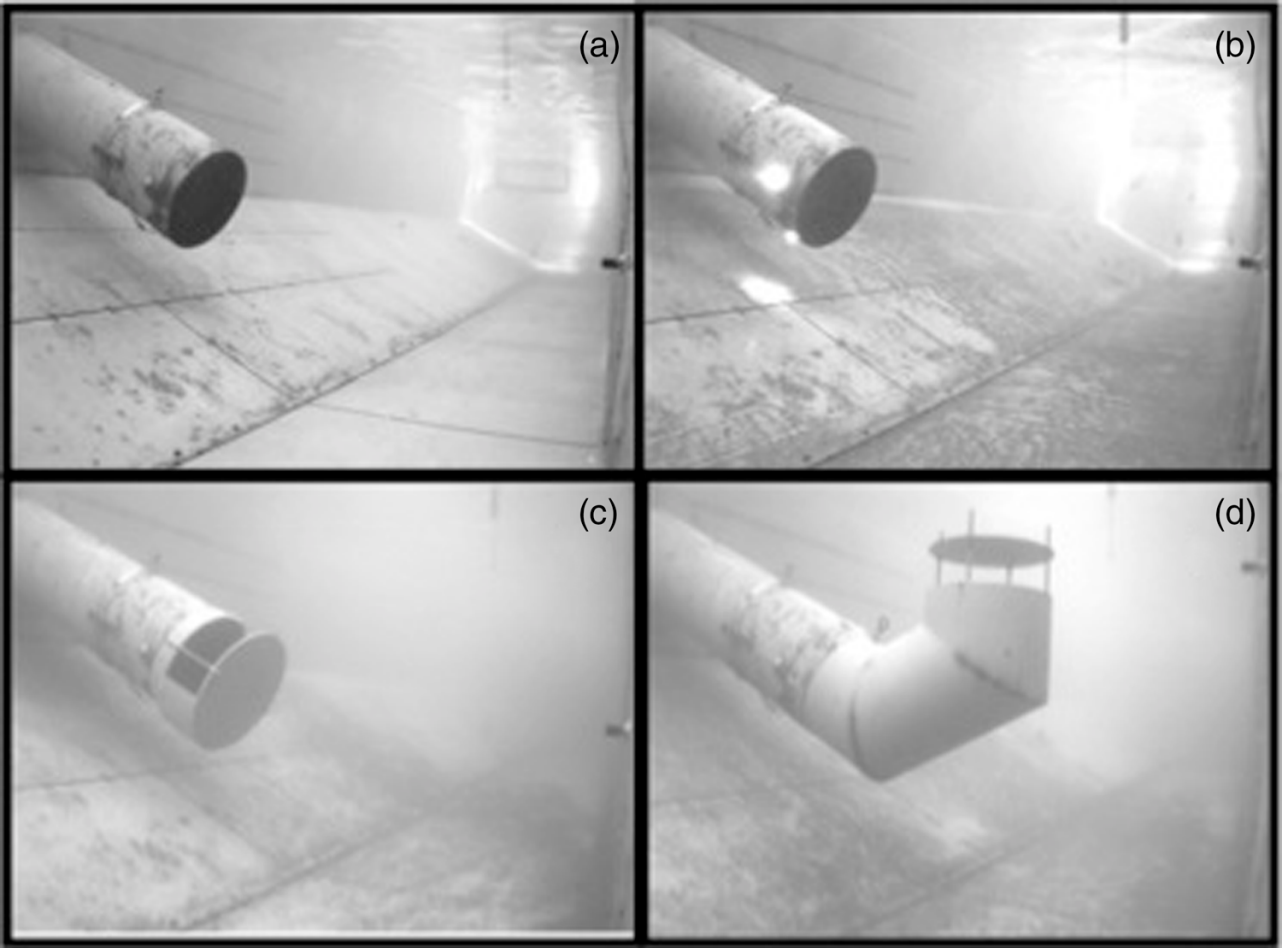
Images of the sensory deterrent and pipe modifications used in experimental trials. Images were taken from an underwater camera downstream of the diversion pipe. (**a**) Control treatment, in which no modifications were made to the pipe. (**b**) Strobe light treatment, in which four strobe lights were placed on the outside circumference of the pipe inlet. (**c**) Terminal pipe plate (TPP) treatment, in which a steel plate and partial steel collar were fitted to the pipe. (**d**) Upturned pipe (UTP) treatment, in which sections of additional pipe were affixed to the pipe inlet to alter the orientation of the intake, and a steel plate was fitted to the pipe opening.

The efficacy of a sensory deterrent or structural pipe modifications in reducing the entrainment of juvenile green sturgeon was tested in a large (>500 kl), outdoor, rectangular, recirculating flume with a testing area that was 18.29 m long, 3.05 m wide and 3.20 m deep (for flume specifications, see Mussen *et al.*, 2013). A 46-cm-diameter diversion pipe was located along one wall of the flume at approximately one-half the length of the flume and projected into the flume at an angle of 26.6° to simulate a typical ‘over-the-levee’ style diversion found in the Sacramento River (Fig. 1a). The flume walls and diversion pipe were constructed out of painted steel, and the floor of the flume was constructed of reinforced concrete. The flume was designed to keep the hydraulic pumps electrically isolated from the water to minimize stray electrolysis, and we observed no abnormal or erratic behaviour in the sturgeon. The sweeping or ‘river’ water velocity through the flume was maintained at 15 cm s^−1^, and the volume of water diverted through the diversion was maintained at 57 cm^3^ s^−1^ for all treatment conditions. This combination of sweeping flow and diversion rate is within the range of typical operational flows (Dan Meier, United States Fish and Wildlife Service, personal communication), has been shown to entrain high numbers of juvenile green sturgeon in previous experiments, and allowed for comparisons with other native California fish species tested using similar methodologies (i.e. Chinook salmon; Mussen *et al.*, 2014b).

We tested juvenile green sturgeon between late August and mid-September 2011 during the day, using the hydraulic conditions described above, in four different treatment conditions: control, strobe light, terminal pipe plate (TPP) and upturned pipe (UTP). In control conditions, the diversion pipe was left open and unaltered (Fig. 1a). For the strobe light treatments, four LED strobe lights (Rotan, QuasarDot) were positioned around the outer periphery of the diversion pipe at 0, 90, 180 and 270°, 14 cm from the end of the pipe intake (Fig. 1b). Each strobe light emitted four rapid pulses of light over 0.5 s, with a 0.5 s pause before the next flash cycle. In TPP treatments, a circular steel plate 52 cm in diameter and 2 cm thick was affixed 15.2 cm from the pipe inlet using four threaded metal rods. A 2-cm-thick steel collar was also attached between the pipe inlet and the steel plate on the bottom half of pipe intake (Fig. 1c). This design reduced the intake velocities directly in front of and below the pipe intake by distributing the velocities around the circumference of the pipe, while still maintaining an overall diversion rate of 57 cm^3^ s^−1^. A similar steel plate without the additional partial collar was used in the UTP treatment, where an additional 97.7 cm of flow path was added to the pipe in two sections with 58° angle bends. This altered the position of the pipe inlet vertically in the water column (Fig. 1d). Water depth was maintained at 2.2 m for control, strobe light and TPP treatments. The water depth necessary to achieve the same 57 cm^3^ s^−1^ diversion rate through the pipe was 2.6 m for the UTP condition.

For each experimental trial, 60 (±3) naïve juvenile green sturgeon that had no prior experience in the flume were tested. Fish were 34.9 ± 0.3 cm in total length (TL; mean ± SEM), weighed 162.9 ± 4.0 g (±SEM) and were 128–141 days post-hatch in age. Six trials were conducted for each treatment, and each trial lasted 1 h. Fish diverted through the pipe were collected, weighed and measured separately from fish that remained within the flume. Water temperature and dissolved oxygen at the start of experimental trials were 19.8 ± 0.2°C and 7.3 ± 0.2 mg O_2_ l^−1^ (means ± SEM), respectively, and 20.5 ± 0.2°C and 7.4 ± 0.2 mg O_2_ l^−1^, respectively, at the end of experimental trials. Additional details of the experimental procedure followed those of Mussen *et al.* (2014a).

Underwater cameras (Speco CVC 320) were positioned within the flume to record fish behaviour near the diversion pipe. Three cameras were positioned on the flume wall across from the pipe inlet; one was mounted directly across from the centre of the pipe inlet, and two were mounted laterally to the pipe inlet, one on each side. A fourth underwater camera was positioned 2.1 m downstream of the diversion pipe on the opposite flume wall. A fifth camera (Sony CCD-TRV 108) was used in combination with a clear Plexiglass acrylic 1.2 m^2^ view plate to provide a direct overhead view of the centre of the pipe intake. Videos were analysed using a video editor (Sony Movie Studio 10).

Several behavioural indices related to entrainment and fish passage were quantified. The number of fish that were entrained through the diversion pipe, the timing of each entrainment event and the distance from which a fish was swimming from the pipe inlet at the time of entrainment were quantified. Entrainment distances were calculated for the first 10 fish entrained in each trial as the resultant distance measured from the centre of the pipe inlet on the plane of the pipe opening to the location of the start of the entrainment event, determined by changes in body position or velocity. For further descriptions of entrainment distance measurements, see Mussen *et al*. (2013). Escape behaviours once an entrainment event began were also noted, and successful escapes where the fish avoided entrainment were quantified. The total number of passes fish made past the pipe was quantified as the number of times fish moved from downstream to upstream or upstream to downstream of the diversion pipe, at any distance from the pipe inlet, regardless of orientation. As we were unable to identify and track individual fish within the flume, the number of total pipe passes was quantified for the group of fish for a given trial; individual rates of passage could not be measured. The timing of fish passage events was also quantified. The proportion of fish entrained for each trial was calculated as the number of fish that were diverted through the pipe divided by the total number of fish that were tested within the flume. The entrainment risk per pipe passage (EPP) was calculated for each trial as the total number of entrainment events divided by the total number of times fish moved past the pipe. This is a measure of the risk of an individual fish becoming entrained into the pipe after a single movement past the pipe. The estimated percentage of migrating juvenile green sturgeon lost to entrainment following repeated encounters with diversion pipes was calculated using equation (1) below, where *E* is the estimated percentage of the population lost to entrainment, EPP the entrainment risk per pipe passage and *n* the number of diversion pipes encountered.

(1) $$E=100\times [1-(1-\mathrm{EPP}{)}^{n}]$$

### Data analysis

Data were analysed using the R Studio version 2.15.2 software package (R-CoreTeam, 2012). Statistical analyses in R were performed using the *R core package* (R-CoreTeam, 2012), ‘*car*’ (Fox and Weisberg, 2011), ‘*multcomp*’ (Hothorn *et al.*, 2008) and ‘*lme4*’ packages (Bates *et al*., 2014). The proportion of total fish that were entrained and the EPP were analysed using individual generalized linear models, because the residuals were not normally distributed for either metric, and data for the EPP were not homoscedastic. Both behavioural measurements were analysed using a quasibinomial distribution with a logit link function, with ‘treatment’ as a categorical predictor variable with four levels. Subsequent *post hoc* tests were conducted using multiple comparisons for parametric models with single-step adjusted *P* values to make multiple comparisons among treatment levels. Fish mass and total length were compared among treatments and removal location (i.e. diverted through the pipe or remaining in the flume) using a two-way analysis of variance of ‘treatment’ and ‘location’, and a generalized linear model of the same predictor variables using a γ distribution with an inverse link function, respectively. The number of entrainments over time was analysed using a generalized linear mixed model using a Poisson distribution and an offset term (logarithm of the total number of fish entrained), with ‘treatment’ and ‘time’ as fixed effects. ‘Time’ was a categorical variable with six levels: 10, 20, 30 min into the trial, etc. Time within treatment within each trial, trial number within treatment, and trial number were all considered random effects. The total number of fish passages and the distances from which fish were entrained were each analysed using a one-way analysis of variance with subsequent Tukey's *post hoc* tests to compare among treatment groups. The number of pipe passages over time was analysed using a two-way analysis of variance, with ‘time’ and ‘treatment’ as variables, and subsequent Tukey's *post hoc* tests. ‘Time’ was a categorical variable with six levels: 10, 20, 30 min into the trial, etc. The number of successful escape attempts per number of entrainments was quantified for each trial and analyzed using a Kruskal–Wallis one-way analysis of variance on ranks. Statistical significance was considered at α ≤ 0.05.

## Results

### Fish size

There were no significant differences in the mass of the fish among treatments (*F*_3,42_ = 2.63, *P* = 0.063) or between entrained and non-entrained fish (*F*_1,42_ = 1.65, *P* = 0.21). Treatment and retrieval location (flume vs. diverted) were also not significant predictors of fish TL (*P* = 0.13, *P* = 0.22, respectively).

### Total pipe passages

There were no significant differences among treatments in the total number of times that fish passed the diversion pipe (*F*_3,20_ = 1.35, *P* = 0.29). In control conditions, fish swam past the pipe a mean of 108.8 (±12.5, SEM) times. Likewise, fish swam past the pipe 118.7 (±17.5, SEM) times under strobe light conditions, 142.0 (±16.2, SEM) times during TPP treatments and 94.8 (±21.1, SEM) times during UTP treatments.

### Pipe passages over time

There was a significant effect of time on the number of times that fish swam past the pipe (Table 1; *F*_5,120_ = 7.27, *P* = 5.6 × 10^−6^), but no significant interaction between treatment and time (*F*_15,120_ = 1.33, *P* = 0.19). Significantly more fish passed the pipe during the 20–30, 30–40 and 40–50 min time periods than those that passed from 0 to 10 min (*P* = 2.1 × 10^−5^, 4.8 × 10^−5^ and 0.02, respectively), and significantly more fish swam past the pipe during the 20–30 min period than those that passed during the 10–20 min time period (*P* = 0.03).

**Table 1:** Mean (±SEM) number of entrainments and total fish passages for each treatment for a given 10 min time period during 1 h experimental trials (*n* = 6 for each treatment)

Time period (min)	Treatment							
	Control		Strobe light		TPP		UTP	
	Entrainment	Passage	Entrainment	Passage	Entrainment	Passage	Entrainment	Passage
0–10	3.8 (±1.3)	12.7 (±3.3)	3.8 (±1.1)	15.7 (±3.8)	0.5 (±0.2)	9.5 (±1.1)	0.2 (±0.2)	4.5 (±2.1)
10–20	4.0 (±1.0)	19.5 (±3.6)	6.5 (±1.3)	21.3 (±3.4)	0.3 (±0.2)	17.5 (±2.9)	0.0 (±0.0)	8.3 (±2.7)
20–30	5.7 (±1.2)	27.0 (±4.1)	8.8 (±1.4)	27.7 (±5.7)	2.2 (±0.4)	28.5 (±4.7)	0.2 (±0.2)	19.5 (±4.8)
30–40	6.0 (±1.0)	22.2 (±3.6)	3.7 (±1.1)	21.3 (±6.0)	2.2 (±0.5)	32.5 (±3.0)	0.3 (±0.2)	24.3 (±3.7)
40–50	2.8 (±0.4)	14.0 (±3.8)	6.0 (±2.3)	19.0 (±7.4)	1.5 (±0.3)	31.8 (±2.0)	0.5 (±0.3)	16.7 (±3.6)
50–60	2.5 (±0.8)	13.5 (±5.1)	2.2 (±0.7)	14.0 (±3.3)	0.5 (±0.3)	22.2 (±6.0)	0.8 (±0.5)	21.3 (±5.3)

There was a significant effect of treatment (*P* < 0.05) and time (*P* < 0.05) and a significant interaction between the two (*P* < 0.05) on the number of entrainment events. There was a significant effect of time (*P* < 0.05) on the total number of fish passages. Entrainment over time was analysed using a generalized linear mixed model, and details of the significance of the factors can be found in the Results section. Passage over time was analysed with a two-way analysis of variance and subsequent Tukey's *post hoc* tests, and the results of the *post hoc* tests are described in the Results section. Abbreviations: TPP, terminal pipe plate; and UTP, upturned pipe.

### Proportion of fish entrained

**Figure 2:**
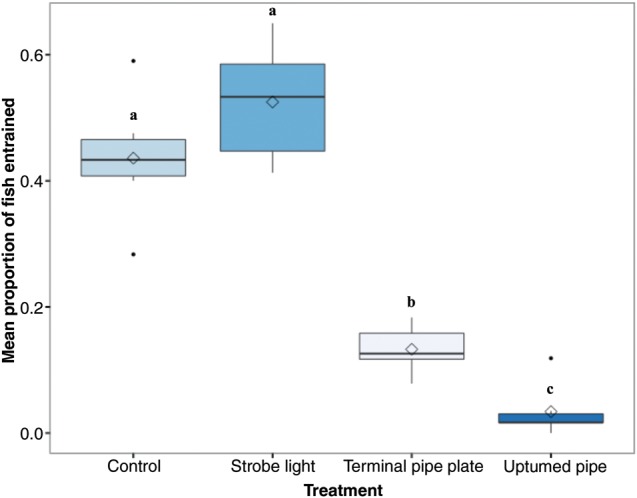
The proportion of fish entrained into the diversion pipe for each treatment. Boxplots of the proportion of fish that were diverted through the diversion pipe in each trial out of the total number of fish tested within the flume. Different letters represent statistically significant differences among treatments. Key: black line, median; box, interquartile range; whiskers, 1.5 × interquartile range; filled circles, outliers; and open diamond, mean. Mean proportions of fish diverted for each treatment (±SEM) are reported in the text. *n* = 6 trials for each treatment; 60 (±3) fish per trial.

The deterrent treatment used was a significant predictor of the proportion fish entrained through the diversion pipe (*P* < 2.2 × 10^−16^, d.f. = 3), and there were significant differences between treatments (Fig. 2). The strobe light treatment entrained the greatest proportion of fish (0.53 ± 0.04; mean ± SEM), though this was not significantly different from the entrainment by the control treatment (0.44 ± 0.04). The TPP treatment entrained the second lowest proportion of fish through the diversion pipe (0.13 ± 0.02), and the UTP treatment entrained the lowest proportion of fish (0.03 ± 0.02).

### Entrainment risk per pipe passage

**Figure 3:**
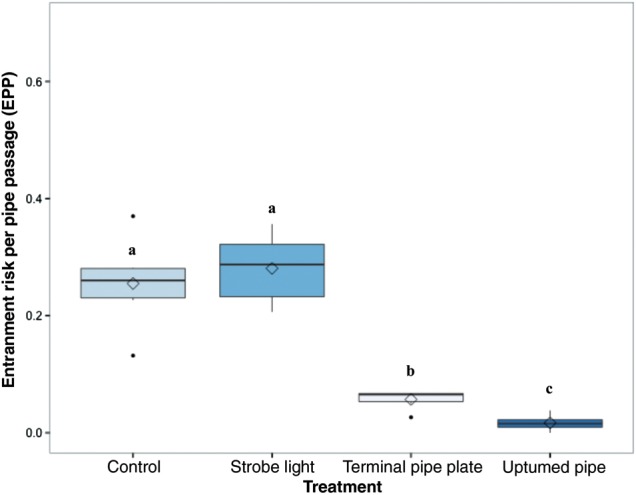
Entrainment risk per pipe passage (EPP) for each treatment. Boxplots of the risk of an individual fish becoming entrained after passing the diversion pipe a single time, moving either upstream or downstream. Different letters represent statistically significant differences among treatments. Key: black line, median; box, interquartile range; whiskers, 1.5 × interquartile range; filled circles, outliers; and open diamond, mean. Mean proportions of fish diverted for each treatment (±SEM) are reported in the text. *n* = 6 trials for each treatment; 60 (±3) fish per trial.

Treatment was found to be a significant predictor of the risk for an individual fish to become entrained after passing the diversion pipe a single time (*P* < 2.2 × 10^−16^, d.f. = 3), and there were significant differences in the EPP between treatments (Fig. 3). The strobe light treatment had the highest EPP value (0.28 ± 0.02; mean ± SEM), but this was not significantly greater than the risk posed by the control treatment (0.25 ± 0.03). The second lowest EPP was posed by the TPP treatment (0.06 ± 0.01), and the UTP treatment had the smallest EPP (0.02 ± 0.01).

### Entrainment over time

There was a significant effect of time on the number of fish that were entrained (*F* = 4.7, *P* = 0.006), a significant effect of treatment (*F* = 31.7, *P* = 1.57 × 10^−12^) and a significant interaction between the two (*F* = 1.7, *P* = 0.001). A greater number of fish were entrained at 30 and 40 min into the trial, and this difference in entrainment events over time was more pronounced for the control and strobe light treatments compared with the two structural modifications (Table 1).

### Successful escape attempts

The number of successful escape attempts relative to the number of entrainment events was not significantly different among treatments (χ^2^ = 1.4, d.f. = 3, *P* = 0.72). While not significantly different, the number of successful escape attempts per fish entrained was greater for the TPP and UTP treatments (0.12 ± 0.08 and 0.20 ± 0.18, respectively; mean ± SEM) than for the control and strobe light treatments (0.07 ± 0.03 and 0.10 ± 0.04, respectively).

### Entrainment distance

**Figure 4:**
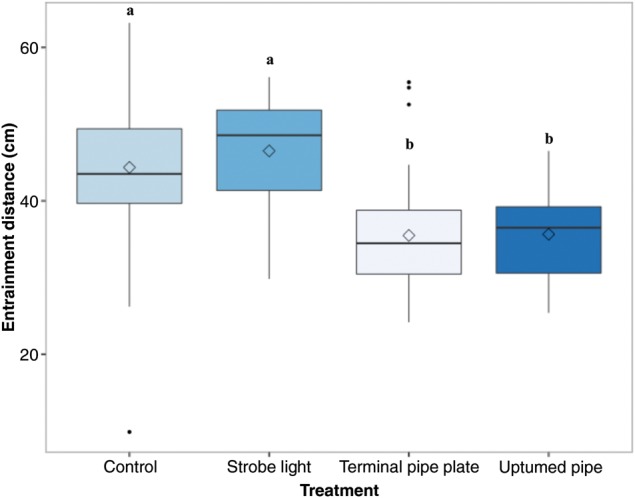
The resultant distance from which fish were entrained into the diversion pipe. Boxplots of the distance from the centre of the pipe inlet that fish were swimming when an entrainment event began. Different letters represent statistically significant differences among treatments. Key: black line, median; box, interquartile range; whiskers, 1.5 × interquartile range; filled circles, outliers; and open diamond, mean. Mean proportions of fish diverted for each treatment (±SEM) are reported in the text. *n* = 6 trials for each treatment; 60 (±3) fish per trial.

The distance from the centre of the pipe to where fish entrainments began was significantly different among treatments (Fig. 4; *F*_3,171_ = 22.8, *P* = 1.92 × 10^−12^). Both the control and strobe light treatments entrained fish from a significantly greater distance from the centre of the pipe inlet in comparison to the TPP and UTP treatments (44.4 ± 1.1 and 46.5 ± 0.9 cm, respectively, vs. 35.5 ± 1.1 and 35.6 ± 1.7 cm, respectively).

### Estimated entrainment

**Figure 5:**
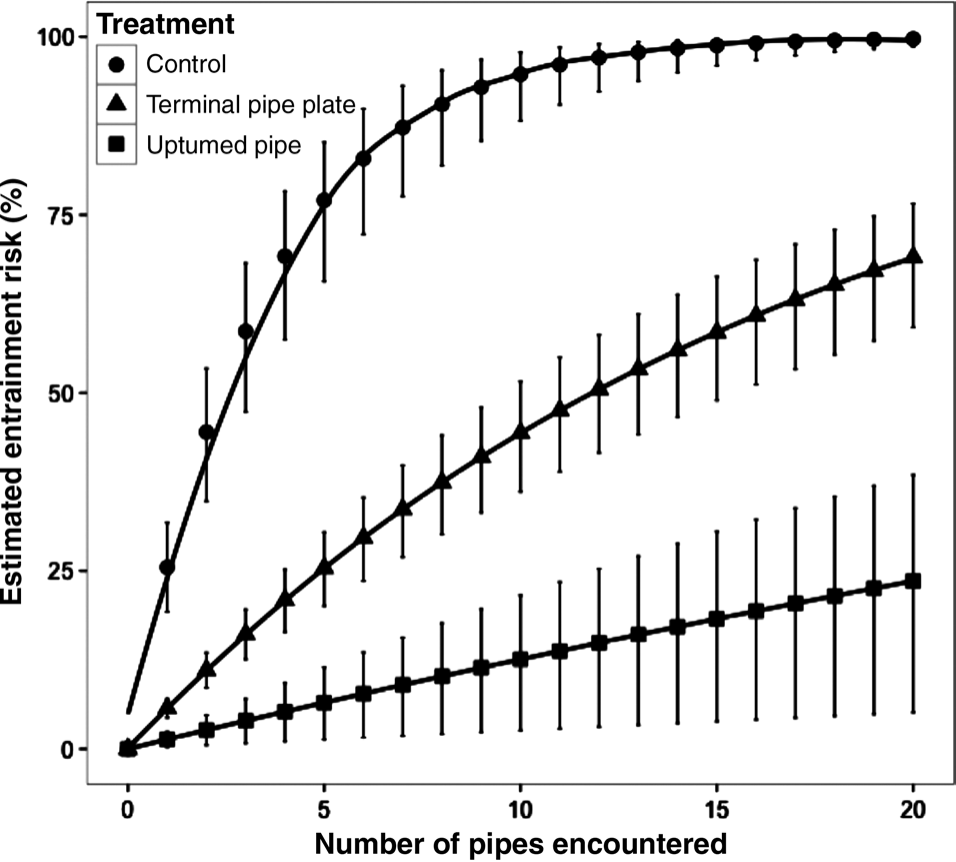
The estimated entrainment risk for juvenile green sturgeon after multiple encounters with diversion pipes. The estimated entrainment risk was projected from the entrainment risk per pipe passage (EPP) values obtained during experimental trials for the two treatments that significantly reduced EPP compared with control conditions. Vertical black bars represent 95% confidence intervals.

Using the EPP rates obtained from experimental trials, we estimated the potential entrainment risk of outmigrating juvenile green sturgeon following repeated encounters with active unscreened diversion pipes (Fig. 5). We made these estimates under the assumption of no learning on the fish's part after the initial encounter, and using data obtained at one set of river conditions in a flume with a fixed width of ∼3 m. In control conditions, up to 58.6% of migrating juvenile green sturgeon could potentially become entrained after passing only three diversion pipes. This number dropped to 16.1 and 4.0% when the TPP or UTP modifications, respectively, were added to the pipe inlet.

## Discussion

The results obtained from our assessment of methods to reduce entrainment of juvenile green sturgeon into water diversions suggest that effective deterrent designs are possible. Both structural modifications significantly decreased the number of green sturgeon that were diverted through the pipe and resulted in a much lower entrainment risk. The structural modifications were significantly more successful at decreasing entrainment than was the sensory deterrent we tested (strobe lights), which did not significantly alter entrainment compared with the unmodified control conditions. Given that the results obtained in our laboratory swimming flume approximate river flows in one set of field conditions, we do suggest that caution should be used in assuming that our modifications would yield similar successful results for other water-diversion structures, flow conditions, tidal effects and fish species.

Overall, the control conditions entrained a relatively high proportion of juvenile green sturgeon, which has been documented previously in a variety of hydraulic conditions (Mussen *et al.*, 2014a). Nearly 44% (range 28–59%) of fish tested were entrained through the diversion pipe in control conditions. Although numbers of green sturgeon entrained in the wild are largely unknown and difficult to compare with results obtained in the laboratory, our results nonetheless underscore the potential threat that unscreened water diversions pose to this threatened fish species, and highlight the need for effective management strategies.

The total number of times that green sturgeon swam past the pipe, the proportion of fish in the flume that were diverted through the pipe, the EPP and the distance from which fish were entrained were not different for fish tested in the presence of strobe lights and those tested with an unmodified, open pipe. The use of strobe lights has been successful at deterring other species of fish near anthropogenic devices, such rainbow smelt (*Osmerus mordax*; Hamel *et al.*, 2008), but their effect on individual species in various environmental conditions has proved difficult to predict. For example, water flow velocity has been shown to modify the response of white perch (*Morone americana*) and spot (*Leiostomus xanthurus*) to strobe lights (Sager *et al.*, 2000), while Chinook salmon (*O. tshawytscha*) exhibited aversion to strobe lights for only a short period of time (∼40 min) when tested as a sensory deterrent in the laboratory (Mussen *et al.*, 2014b). The inclusion of strobe lights to augment the efficacy of aversive sensory devices may be successful at increasing avoidance responses, and some evidence exists in support of their inclusion in multimodal deterrents (Sager *et al.*, 2000), although recent evidence seems to refute this claim (Ruebush *et al.*, 2012).

The lack of behavioural avoidance by green sturgeon in response to strobe lights shown here is consistent with a previous study, in which neither juvenile green nor white sturgeon (150–198 days post-hatch) behaviour near fish-exclusion screens was altered in the presence or absence of strobe lights in an indoor swimming flume (Poletto *et al.*, 2014). Given that most sturgeon are generally benthic foragers and not visual predators (Moyle, 2002), it is perhaps unsurprising that they are not well adapted to respond to visual stimuli, such as strobe lights. The green sturgeon retina is dominated by rods as the primary photoreceptors, indicating that they are adapted to scotopic environments characterized by low light levels (Sillman *et al.*, 2005). An analysis of the retinal topography of green sturgeon by Sillman *et al*. (2005) also revealed that the retinal rod density of green sturgeon was much lower than that for other animals adapted to low-light environments and was even nearly half as dense as the retina of the channel catfish (*Ictalurus punctatus*), which occupies a similar niche (Sillman *et al.*, 1993). This indicates that green sturgeon vision is likely not to be particularly sensitive or acute (Sillman *et al.*, 2005), and similar results have been found for other *Acipenser* species (Sillman *et al.*, 1999, 2005). The use of strobe lights as a behavioural deterrent for sturgeon, therefore, is likely not an effective means by which to manage sturgeon populations near anthropogenic devices. This conclusion underscores the importance of empirical evaluations of the sensory capabilities of the targeted fish species prior to the implementation of management practices.

The TPP and UTP structural modifications tested here both significantly decreased entrainment of juvenile green sturgeon compared with that of the unmodified control pipe. Neither structural amendment decreased the total number of times fish swam past the pipe, indicating that the fish in the flume were equally exposed to the treatments. Despite the similar pipe passage frequencies, both the TPP and UTP treatments entrained a significantly smaller proportion of green sturgeon than the control treatment, resulting in the decreased EPP rates. Additionally, fish in each pipe modification treatment became entrained into the diversion pipe at a closer distance than those in the unmodified control pipe, further underscoring the differences among the treatments.

While the total water-diversion intake rate remained the same for each of the treatment conditions, the TPP and UTP altered flows near the pipe inlet. The addition of the plate and partial collar on the bottom of the pipe for the TPP treatment resulted in a redistribution of the maximal intake velocity over a greater area. The resulting decrease in intake velocities could have resulted in an increased potential for fish to escape entrainment by exhibiting burst swimming and escape behaviour. While the number of successful escape attempts per entrainment event was slightly greater for both of the structural modifications, this difference was not statistically distinguishable; therefore, it is unlikely that the mechanism by which the TPP reduced entrainment could be attributed to swimming behaviour successfully overcoming intake velocities and allowing fish to avoid entrainment.

The redistribution of the intake velocity caused by the addition of the UTP resulted in the dispersion of water flow over a larger area compared with that of the control condition (Table 2). The highest flow velocity in the *y* (width of the flume) and *z* (depth of the flume) directions changed from 24.4 cm s^−1^ measured 38.1 cm upstream of the diversion pipe to 221.6 cm s^−1^ measured at the diversion pipe for the control conditions. The same measurements taken for the UTP conditions changed from 37.2 to 67.2 cm s^−1^. This effectively created a large, though diminished, disruption in the laminar flow of water around the diversion pipe. This larger area of disruption could have extended the hydrologic stimuli of the pipe further from the centre of the pipe intake, allowing for detection of the diversion pipe by fish at an extended distance. The lateral line of green sturgeon (the system of superficial and canal neuromasts used to detect particle motion in the water surrounding the body of the fish) is less extensive than that of salmonids (J. B. Poletto and D. E. Cocherell, unpublished data) and may therefore be less sensitive to changes in water movement. A recent morphological investigation into the distribution of lateral line receptors (canal and superficial neuromasts) in Siberian sturgeon (*Acipenser baerii*) discovered fewer lines or branches of receptors compared with those of other fish species (Song and Song, 2012), although whether this results in a diminished sensitivity to water movement remains speculative. In previous studies of green and white sturgeon near fish screens, mechanical vibration of the screens themselves failed to alter the behaviour of either species significantly, indicating that particle motion at 10 Hz either was not detected by these fish or was not sufficiently aversive (Poletto *et al.*, 2014). Expanding the distance over which the water velocity changes, as was the case for the UTP, would result in a more gradual water velocity gradient that may provide sturgeon with an opportunity to detect the change in water velocity and avoid the diversion pipe. Likewise, reducing the total change in the water velocities of the sweeping flow relative to the diversion flow, as was the case for the TPP, might allow sturgeon to pass the pipe before the intake velocities threaten to overwhelm their swimming capabilities. These more gradual changes in flow velocity may also assist other fish species in avoiding entrainment by providing opportunities for burst swimming away from the diversion.

**Table 2:** The fastest resultant velocity in the *y–z* axis at a given *x* position within the flume for each treatment that significantly decreased entrainment of juvenile green sturgeon

Relative *x* position (cm)	Peak resultant velocity *y–z* axis (cm s^−1^)		
	Control	TPP	UTP
−76.2	10.1	5.7	19.2
−38.1	24.4	20.9	37.2
0	221.6	4.8^a^	62.2
38.1	38.1	30.6	21.3
76.2	15.9	11.5	11.6

The relative *x* position is the position along the length of the flume relative to the diversion pipe; *x* < 0 is upstream of the diversion pipe, *x* = 0 is the location of the diversion pipe, and *x* > 0 is downstream of the pipe. The peak resultant velocity in the *y–z* axis is the fastest velocity measured at a given *x* position in the flume in the *y* (width) and *z* (depth) axes.

^a^This measurement was taken in the main flow channel, not between the pipe plate and the centre of the pipe inlet as was done for the UTP.

Changes in water flow velocity, such as areas of strong flow acceleration, can act as an additional type of sensory deterrent for fishes. Wild-caught naturally migrating juvenile Chinook salmon avoided areas with rapidly accelerating water flow when tested in an experimental flume and displayed swift changes in swimming behaviour upon approaching the area of acceleration (Enders *et al.*, 2009). Likewise, four species of naturally migrating Pacific salmon smolts avoided an area of rapid acceleration of water flow when presented with a route selection choice in an experimental flume (Kemp *et al.*, 2005). Behavioural avoidance of these areas is thought to be an adaptive response that prevents smolts from entering passage routes that may be unsuitable or dangerous for continued migration, and could reduce the risk for predation that might occur if the fish is left disoriented following movement through such an area (Enders *et al.*, 2009). To our knowledge, behavioural responses to areas with rapid water acceleration have not been specifically investigated in sturgeon species. However, given the poorer swimming capabilities (Peake *et al*., 1997; Adams *et al*., 1999) and unique morphology of Acipenseriforms (Webb, 1986; Peake *et al*., 1997), it seems possible that these fish would likewise avoid areas of accelerating water velocity so as to prevent injury or disruptions in downstream migrations. Therefore, the large areas of more gradual water velocity gradients created by the structural modifications (still resulting in flow acceleration) may have assisted in preventing sturgeon from swimming near the diversion pipe, thus decreasing the risk for entrainment.

The significant differences in distance from which fish were entrained between both structural modifications and the control conditions also underscore the efficacy of these deterrents. Entrainment distances were reduced from roughly 45 and 46 cm for the control and strobe light treatments, respectively, to roughly 35 cm for both the TPP and UTP treatments. This reduction in entrainment distance indicates that juvenile green sturgeon were significantly closer to the pipe inlet before becoming entrained, probably decreasing the effective encounter rate of sturgeon with the pipe and contributing to the lower risk of entrainment observed for these two treatments.

An additional indication that the two structural modifications affected juvenile green sturgeon in a different manner to the control and strobe light conditions is the difference in the number of entrainments over time among the treatments. There was a significant main effect of time on the number of entrainments and also a significant interaction between time and treatment. While the effect of time on entrainment events was significant, we believe this to be an artifact of the large size of the experimental flume. Given that the fish were introduced into the flume upstream of the diversion pipe, the increase in the number of entrainment events observed between 20–30 and 30–40 min into the trials likely reflects the time it took for the majority of the fish to travel downstream towards the diversion pipe, because the number of fish passages also increased during these time periods. The effect of time was also significant on the total number of times that fish moved past the diversion pipe. The significant interaction between time and treatment on the number of entrainments, but not on the number of fish passages, however, indicates that the differences in entrainment over time for the different treatments may reflect a true change in the behaviour of the fish. The timing of entrainment events was more consistent for the two structural modifications, which exhibited a much more modest increase in entrainment during the middle of the trial in comparison to the control and strobe light conditions; however, this also may be due to the much lower number of entrainments that occurred for the structural modifications. Additional research into the effect of time spent near diversion pipes on the behaviour of juvenile green sturgeon is needed before more accurate predictions about the effect of deterrents over time can be made.

From the data obtained in our experimental flume, we projected the estimated entrainment of migrating juvenile green sturgeon after repeated encounters with water diversions when water-diversion pipes were left unmodified (control) and when they were fitted with the two structural modifications (Fig. 5). These estimates were made under the assumption that fish were not altering their behaviour after their initial encounter, and were based on laboratory-obtained data gathered in only one set of flow conditions in a flume with a constant width (∼3 m). These values are intended to represent the upper limit of entrainment estimates, and it is likely that the true values observed in the wild will vary. While these estimates are quantitative, we intend them to demonstrate qualitatively the risk that water diversions can pose and to emphasize the exponential nature of their potential impact. Based on our data, after passing within roughly ∼3 m of only three unmodified water-diversion pipes, up to nearly 59% of migrating juvenile green sturgeon are at risk of becoming entrained. This number increases to nearly 75% when fish pass five pipes. These results underscore the risk posed by these water diversions to migrating juvenile fish and highlight the need for successful mitigation practices.

Despite displaying the lowest proportion of fish entrained and the smallest entrainment risk, we feel that the UTP treatment may not be the most feasible solution to the problem of fish entrainment into open water diversions. In comparison to the TPP structural modification, the UTP was difficult to install, and the resultant increase in the height and size of the water diversion may limit the number of diversion pipes to which it could be affixed. For example, the UTP could be installed only on pipes located in waterways with sufficient depth to prevent the pipe from breaching the surface, and the increased size could become a possible navigation hazard for passing boats. Furthermore, the change in the orientation of the pipe intake, though significantly reducing the risk of entrainment for benthic species that do not spend the majority of the time in the upper portions of the water column, could introduce or exacerbate the risk for pelagic species, such as delta smelt. Therefore, we recommend that modifications similar to the TPP used in our experimental flume have the potential to reduce entrainment while still allowing for sufficient water-diversion rates.

Overall, our results demonstrate that effective management strategies aimed at decreasing the entrainment of juvenile green sturgeon (and probably additional California native fish species) can be reconciled with agricultural and urban water use demands. The two structural modifications tested in our large-scale river simulation flume not only significantly decreased the proportion of fish that were entrained, but also diverted the same rate of water as an unmodified pipe. The risk of entrainment per pipe passage was also significantly decreased by the use of the structural modifications, which resulted in a much lower projected entrainment risk for outmigrating juvenile green sturgeon. In contrast, the sensory deterrent tested (the use of strobe lights) did not result in significant reductions in entrainment risk and did not alter the behaviour of passing sturgeon. Therefore, we suggest that empirical investigations into the efficacy of fish-passage devices or sensory deterrents be completed for each target species prior to the implementation of such devices on water diversions. Our results suggest that affordable and effective fish deterrents can be designed when the physiology, ecology, and sensory capabilities of the fish are considered.

## Funding

This work was supported by the California Department of Fish and Wildlife's Ecosystem Restoration Program (Agreement # E0783004 to N.A.F. and J.J.C.) and the University of California Agricultural Experiment Station (grant no. 2098-H to N.A.F.). This research indirectly benefited from funding from the US Department of Interior's Anadromous Fish Screen Program and the National Science Foundation Graduate Fellowship Program (grant to J.B.P.).
